# Auscultation and Percussion

**Published:** 1854-11

**Authors:** 


### Reviews and Bibliographical Notices.

**AUSCULTATION AND PERCUSSION.** By Dr. Joseph Skoda. Translated from the Fourth Edition by M. O. Markham, M. D., Assistant Physician to St. Mary's Hospital, Philadelphia, Lindsay & Blakiston, 1854. Pp. 380.

We have here the work of the distinguished Skoda, the newness and originality of whose views have at various times caused great excitement in the medical world. Skoda has no respect for old theories and tenets in auscultatory medicine. He ferociously charges into their midst, riding down and destroying, and as Dr. Markham says, "covering the field with the *disjecta membra*" of his luckless adversaries. As may be supposed, the first promulgation of Skoda's views drew upon him the combined wrath of those who assumed to speak for the profession—but although restrained for a time, they have slowly found their way to the minds of men and there wrought conviction, until now the name of Skoda stands in the first rank of the army of medical philosophers. But who will wonder that his views were rejected as dreamy and speculative Germanisms, when he learns that they clasped with and would have set aside the opinions of the venerated and immortal Laennec? Even now when every one acknowledges the accuracy of Skoda's experiments and the truthfulness of his deductions, it is with a feeling akin to horror that we read his attacks upon the fathers of auscultation—but there is a quiet earnestness in his manner of writing that tells that you are dealing with no shallow, brainless theorist, but a philosophical observer and a profound thinker. The limits of a review like the present will not admit of anything like a lengthened exposition of the views of Dr. Skoda, but we will endeavor to place before our readers some of his most prominent ideas. And before entering upon the discussion, we would say that our author deserves the thanks of the profession for his efforts to do away with the hideous, Gallican nomenclature, in which as our own bard Holmes says,

> The bruit de rape and bruit de scie,
> And bruit de diable are all combined,"

for nothing has contributed more to retard the progress of auscultation than the complicated and useless phraseology with which it has been burdened by the French, who, we are sorry to say, find many servile imitators among American physicians who have visited France. Skoda's nomenclature is plain and practical, and would serve an admirable purpose, were it not, as Dr. Markham (the translator) remarks, unfortunately the case that much of it is based upon the theory of consonating sounds, and until that theory is more generally adopted, the nomenclature will not answer. Among its most notable peculiarities are the rejection of ægophony as a sound of no value and of the terms pectoriloquy and broncophony as representing one sound. His division of the dry sounds is interesting, hissing, whistlings and snorings. The most notable of Skoda's theories is that of the broncophonic sounds, which he accounts for by consonance of sound. Our readers will remember that Laennec explains this by supposing that the increased solidity of the parenchyma of the lungs causes the voice to be conveyed with more distinctness to the ear. Skoda's views we will give by transcribing a short sketch of them by Dr. Markham in the translator's preface.

"The voice passes into the parenchyma of the lungs through the medium of the air in the trachea and the bronchial tubes and is not propagated along their walls, it traverses healthy as readily as it does hepatized lung and even somewhat more readily, consequently broncophony does not depend upon an increase of the sound conducting power of consolidated pulmonary tissue, moreover where the lung is consolidated, the thoracic voice increases and diminishes in force without any concurrent change taking place in the condition of the lung, this variation in its strength evidently results from the circumstance of the bronchial tubes being at one moment blocked up by mucus, etc., and at another freed therefrom by the cough and expectoration etc.; if the broncophony depended upon conduction of sound it would be a matter of indifference whether the tubes contained air or fluids. It must not be forgotten that according to the ordinary laws of reflection of sound, the more solid the parenchyma the more difficult does the passage of the sound into it from the air become.

That the air in the mouth and nasal cavities consonates with sounds formed in the larynx, is proved by the fact of the changes which the voice undergoes through opening and closing the mouth and nose, whilst the condition of the larynx remains unaltered, just in the same way does the air in the trachea and bronchial tubes consonate with the laryngeal sounds. Now air consonates only in confined space and the force of the consonance depends upon the form and size of the space and upon the nature of the walls forming it; the more solid the walls, the more completely will the sound be reflected and the more forcible the consonance. The cause of the loud voice produced by a speaking trumpet is well known.—But the air will consonate with certain sounds only; in the trachea and bronchial tubes it becomes consonant with the laryngeal voice, in so far as their walls have a like or analogous character to the walls of the larynx, of the mouth and of the nose. Within the cartilaginous walls of the trachea and the bronchial trunks, the voice consonates nearly as forcibly as in the larynx, but as the bronchial tubes divide in the lungs, they lose their cartilaginous character, becoming at last merely membranous in structure and therefore very poorly adapted for consonance; when therefore, the consonance is increased in these latter tubes, we may be sure, either that the membrane forming them has become very dense or cartilaginous, or that the tissue around them is condensed and deprived of air, whereby the former reflecting power of the tubes is increased. Of course the communication between the air in the tubes and the air in the larynx must be uninterrupted. The walls of a confined space frequently vibrate in unison with sounds excited within it as do those of an organ-pipe or of a speaking trumpet. The larynx vibrates with every sound and its vibrations are perceptible at a considerable distance from their point of origin; so also must the walls of the bronchial tubes which are distributed through the parenchyma of the lungs, vibrate when the voice consonates within them; and the vibrations thus excited, will extend to the surface of the thorax, passing through several inches of thick fleshy parts, or of fluids and manifest themselves there as the consonating sounds of the bronchial tubes.

This then is Skoda's theory of broncophony. Our readers must judge for themselves whether he has established his case.

With an exposition of another of Skoda's theories we will close. We lack space to lay his proofs before the reader, and can only remark that he bases his opinion upon actual experiment.

"That the lungs partially deprived of air should yield a tympanitic sound and when the quantity of air is increased, a non-tympanitic sound appears opposed to the laws of physics. The fact however, is certain and is corroborated both by experiments on the dead dead body and also by this constant phenomena, viz: that when the lower portion of a lung is entirely compressed by any pleuritic effusion, and its upper portion reduced in volume the percussion-sound at the upper part of the thorax is distinctly tympanitic."

"In pneumo-thorax, the walls of thorax, if they are not much distended, yield a tympanitic sound, but if much distended, their sound is almost constantly non-tympanitic."

* * * * "When the intestines contain gas, but are not forcibly distended by it, nor compressed by the abdominal walls, they always render the percussion-sound of the abdomen tympanitic; but if they are much distended or compressed by the muscles of the abdomen, the sound becomes less or even more tympanitic."

The only approach to a just explanation of this position of Dr. Skoda, respecting which at first, there would seem some doubt, would be the fact that the parietal walls would lose their natural contractility in consequence of long and great distention, which would result in a flaccid or *doughy* state of the integuments and therefore would yield, upon percussion, a less resonant sound than in a more contractile and tense condition of the parts. Thus we see that the degree of resonance depends not only upon the amount of enclosed fluids, but also upon the degree of tension of the parietes. Great accumulations of air in a cavity, and at the same time a loss of resonance from the above cause, is always an evidence, in our opinion, of a great loss of vitality, and we cannot well see how it can obtain under other circumstances.

The translator's work in the book has been well performed, with the exception of a tendency to put many of his words into a form too nearly approaching the original German.

Should any one be interested sufficiently in the work to procure it, we can assure them that they will find it deeply interesting, whatever may be their opinion of the truth of the views advanced by its distinguished author.

